# Who’s afraid of DEET? Fearmongering in papers on botanical repellents

**DOI:** 10.1186/s12936-020-03217-5

**Published:** 2020-04-08

**Authors:** Matan Shelomi

**Affiliations:** Department of Entomology, National Taiwan University, Taipei, Taiwan

**Keywords:** DEET, Repellents, Safety

## Abstract

DEET (*N*,*N*-Diethyl-*meta*-toluamide) is considered the gold standard in mosquito repellents, not only for its effectiveness, but also for its safety. DEET has been more extensively studied for safety than any other repellent, and is accepted as completely safe when used correctly (i.e. not consumed or bathed in). Researchers studying botanical repellents, however, often paint DEET as far more toxic than it really is, falsely claiming it is a menace to the public health or even the environment. These claims are unfounded, and often the only evidence given by such publications are references to other publications also studying botanical repellents. Such publications are biased, and may be attacking DEET’s excellent safety record to justify their existence and the need for their research. The inconvenient yet undisputable fact is that no botanical repellent has been proven to be as safe as DEET, and the majority never had any safety testing whatsoever. The automatic assumption that botanical repellents are safer than DEET is the ‘appeal to nature fallacy,’ which also drives most of the market for “natural” repellents, yet natural repellents have side effects and even a body count. Finding a botanical repellent that works as well as DEET and is equally safe is a legitimate research goal on its own, and need not be justified by fear-mongering and irrational chemophobia. Researchers studying these alternatives should strive for integrity, raising the real issue of the lack of safety testing for botanical repellents rather than denying the proven safety of DEET.

## Background

The recent publication by Asadollahi et al. titled “Effectiveness of plant-based repellents against different *Anopheles* species: a systematic review” [1] contains some useful information, but unfortunately, the introduction contains inaccuracies regarding synthetic repellents. The case is one of many papers on plant-based repellents that exaggerate the risks associated with repellents like DEET (*N*,*N*-Diethyl-*meta*-toluamide) that have been demonstrated as safe, while making assumptions about the safety of botanicals without evidence for it or despite evidence to the contrary.

## Main text

Asadollahi et al. state, “it has been identified that chemical repellents are not safe for public health ” [1]. Besides the fact that every repellent, botanical or synthetic, is a chemical repellent, the sole source cited for this claim is a paper by Sanghong et al. [2] promoting the herb *Ligusticum sinense* as “a herbal alternative” [sic] against mosquitoes. That paper not only does not test the safety of “chemical repellents” at all, but also actually states that DEET is “considered safe,” and that the rare, toxic effects attributed to it “have been described mainly after misapplication.” Systematic reviews have confirmed that DEET is safe for humans of all ages when used correctly [3, 4], making Asadollahi’s claim that DEET harms public health both false and unsubstantiated by their own source.

The authors continue by stating, “the frequent use of synthetic repellents with chemical origin for mosquito control has disturbed natural ecosystems and resulted in the development of resistance to insecticides, resurgence in mosquito populations, and adverse impact on non-target organisms”. A repellent is not an insecticide, so this claim is misleading: it is impossible for repellents applied to skin, such as DEET, to affect mosquito populations, impact non-target organisms, or cause resistance to unrelated compounds. The genes for insecticide resistance do sometimes correlate with resistance to repellents, but that applies to natural repellents as well as synthetic ones [5]. Even if Asadollahi et al. were confused about the nature of the repellent-insecticide resistance correlation, there is no basis for them to falsely claim that synthetic repellents differ from natural repellents in this regard. Their sources for the claim are again the paper by Sanghong et al. [2], which does not state anything relating to the matter, and Govindaarajan et al. [6], an article claiming *Zingiber nimmonii* is “an eco-friendly tool” against mosquitoes, and which, while also making debunked or incorrect claims about DEET, clearly states that the issues of ecological disruption are due to insecticides, not repellents.

Asadollahi et al. claim that their systematic review was to identify plant-based repellents that are effective “without causing side effects on human health”. The review however does not provide any information about the safety of any of the plants listed, though likely this is because repellency studies typically do not include tests for safety. Indeed, for most “natural” repellents, there is zero information on safety, yet among non-experts they are automatically assumed to be less toxic than synthetics that have ample, overwhelming evidence to their safety. While the authors do acknowledge that “natural does not equate to safe,” considering their claim to systematically evaluate the safety of natural repellents, it is disappointing that they did not discuss this issue further or cite any of the studies that do exist. Many plant-based repellents are known to be more toxic than DEET at the concentrations necessary to approach its effectiveness, and essential oil poisoning can happen at doses far smaller than for synthetic repellents [7], with both citronella and eucalyptus oil having caused human deaths [8].

Consider formulations containing oil of lemon eucalyptus (OLE) or its active ingredient PMD (*p*-Menthane-3,8-diol), so far the only botanical repellents recommended by the United States Centers for Disease Control and Prevention (CDC) alongside the synthetics DEET, picaridin, IR3535, and 2-undecanone as a safe and effective topical insect repellent [9]. The CDC, the United States’ Food and Drug Administration, the US Environmental Protection Agency, and the American Academy of Pediatrics all agree that the synthetics are safe for infants as young as 2 months, the age below which topical products should probably not be used at all, but forbid using OLE or PMD products on anyone younger than 3 years. The respective justifications are the overwhelming evidence for DEET and the other synthetics’ safety, and the essentially unknown toxicity of OLE. Furthermore, OLE in its pure form is not considered a repellent at all and should not be used [9]. These facts about OLE should certainly have been included in a systematic review of plant-based repellents, but are inconvenient truths for those trying to suggest botanicals are safer than synthetics when, so far, that is objectively not the case.

## Conclusions

The appeal to nature fallacy is the primary driver of the “natural” repellent market and the irrational fear of DEET and other synthetics, as it is for the pseudosciences of homeopathy and the antivaxxer movement. While a natural repellent as safe and effective as DEET but lacking its effect on certain synthetic fabrics (arguably the one genuine drawback to DEET) would be an interesting and commercially viable find, it is unfortunate that so frequently the researchers studying botanical repellents resort to fear-mongering about DEET to justify their work. Better to acknowledge that DEET has the strongest safety record of any repellent on the market, natural or otherwise, but that the ‘appeal to nature fallacy’ amongst consumers is driving the demand for “natural” alternatives, and so research into them continues and should also include appropriate safety testing.

## Data Availability

Not applicable.

## Abbreviations

CDC: Centers for Disease Control and Prevention
DEET: *N*,*N*-Diethyl-*meta*-toluamide
OLE: oil of lemon eucalyptus
PMD: *p*-Menthane-3,8-diol
